# Multidisciplinary treatment of left hepatic artery pseudoaneurysm after hepatobiliary resection for gallbladder cancer: a case report

**DOI:** 10.1186/s40792-019-0757-8

**Published:** 2019-12-09

**Authors:** Ryusei Yamamoto, Teiichi Sugiura, Yukiyasu Okamura, Takaaki Ito, Yusuke Yamamoto, Ryo Ashida, Katsuhisa Ohgi, Takeshi Aramaki, Katsuhiko Uesaka

**Affiliations:** 1 Division of Hepato-Biliary-Pancreatic Surgery, Shizuoka Cancer Center, 1007 Shimo-Nagakubo, Sunto-Nagaizumi, Shizuoka, 411-8777 Japan; 2 Division of Interventional Radiology, Shizuoka Cancer Center, 1007 Shimo-Nagakubo, Sunto-Nagaizumi, Shizuoka, Japan

**Keywords:** Pseudoaneurysm, Hepatectomy, Gallbladder cancer, Thrombosis, Portal vein arterialization

## Abstract

**Background:**

When a postoperative hepatic artery pseudoaneurysm develops after massive hepatectomy, both an intervention for the pseudoaneurysm and patency of hepatic artery should be considered because occlusion of the residual hepatic artery results in critical liver failure. However, the treatment strategy for a pseudoaneurysm of the hepatic artery after hepatobiliary resection is not well established.

**Case presentation:**

A 65-year-old woman underwent right hepatectomy, extrahepatic duct resection, and portal vein resection, for gallbladder cancer. Although the patient had an uneventful postoperative course, computed tomography on postoperative day 6 showed a 6-mm pseudoaneurysm of the hepatic artery. Angiography revealed the pseudoaneurysm located on the bifurcation of the left hepatic artery to the segment 2 artery plus the segment 3 artery and 4 artery. Stent placement in the left hepatic artery was not feasible because the artery was too narrow, and coiling of the pseudoaneurysm was associated with a risk of occluding the left hepatic artery and inducing critical liver failure. Therefore, portal vein arterialization constructed by anastomosing the ileocecal artery and vein was performed prior to embolization of the pseudoaneurysm to maintain the oxygen level of the remnant liver, even if the left hepatic artery was accidentally occluded. The pseudoaneurysm was selectively embolized without occlusion of the left hepatic artery, and the postoperative laboratory data were within normal limits. Although uncontrollable ascites due to portal hypertension occurred, embolization of the ileocolic shunt rapidly resolved it. The patient was discharged on postoperative day 45.

**Conclusion:**

Portal vein arterialization prior to embolization of the aneurysm may be a feasible therapeutic strategy for a pseudoaneurysm that develops after hepatectomy for hepatobiliary malignancy to guarantee arterial inflow to the remnant liver. Early embolization of arterioportal shunting after confirmation of arterial inflow to the liver should be performed to prevent morbidity induced by portal hypertension.

## Background

Curative resection for biliary cancer often requires aggressive surgery with major hepatectomy and dissection of the hepatoduodenal ligament. After surgery, however, the remnant liver function is limited and the oxygen is supplied by only the residual hepatic artery [1]. If a pseudoaneurysm develops after surgery for biliary cancer, both an intervention for the pseudoaneurysm and patency of the hepatic artery should be considered because occlusion of the residual hepatic artery results in severe ischemia of the hepatic parenchyma, resulting in critical liver failure or anastomotic leakage of the choledochojejunostomy [2]. However, the treatment strategy for a pseudoaneurysm after surgery for biliary cancer is not well established.

This case report describes successful multidisciplinary treatment of an aneurysm after hepatobiliary resection for gallbladder cancer.

## Case presentation

**Fig. 1:**
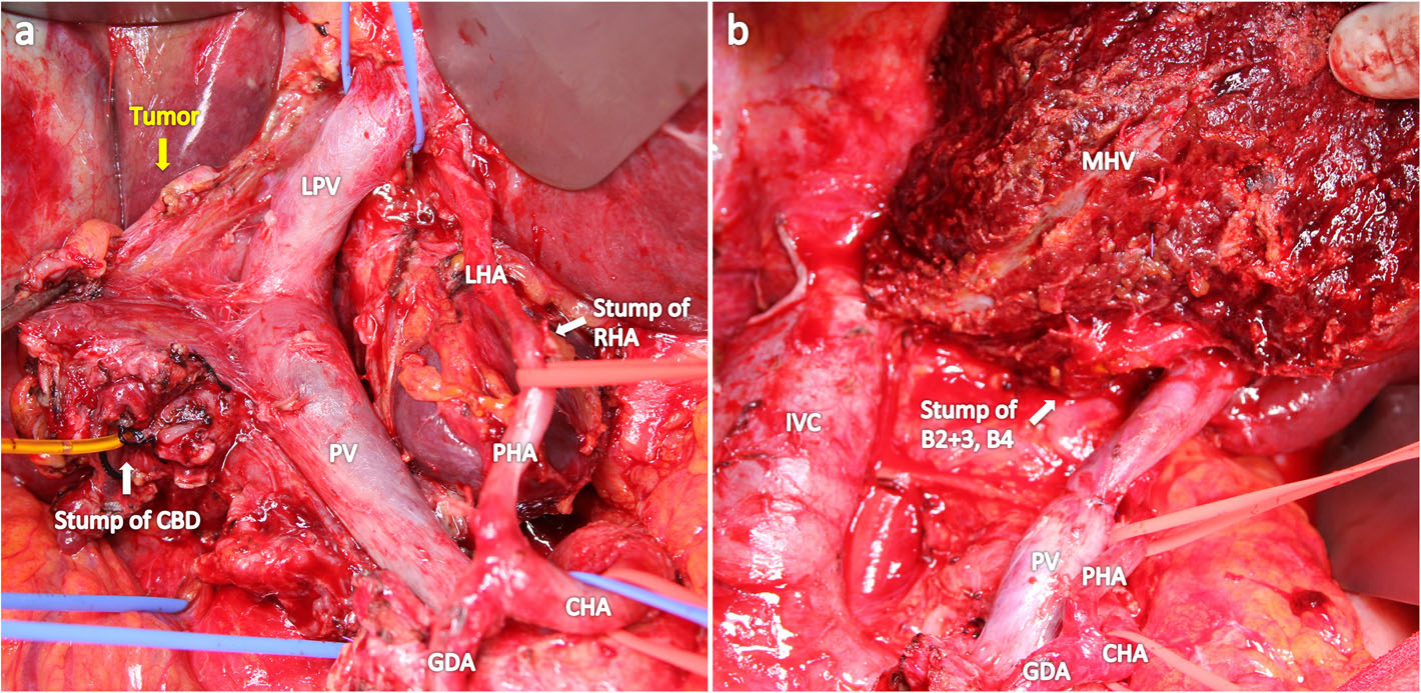
Intraoperative photographs. **a** Gallbladder cancer involving the PV and skeletonized hepatic artery after dissection of the hepatoduodenal ligament. The LHA was exfoliated to the threshold of the hepatic parenchyma, and the bifurcation of the LHA to A2, A3, and A4 was invisible. **b** After the right hepatectomy, extrahepatic duct resection, PV resection, and lymph node dissection were performed. CHA, common hepatic artery; PHA, proper hepatic artery; LHA, left hepatic artery; RHA, right hepatic artery; GDA, gastroduodenal artery; PV, portal vein; LPV, left portal vein; CHA, common bile duct; MHV, middle hepatic vein; IVC, inferior vena cava; A2, segment 2 artery; A3, segment 3 artery; A4, segment 4 artery

A 65-year-old woman with gallbladder cancer was referred to our hospital for surgery. The laboratory examination revealed obstructive jaundice and cholangitis. Computed tomography (CT) showed gallbladder cancer involving the hepatic hilum, including the portal bifurcation. Preoperative cholangitis developed several times, and the endoscopic biliary stent was exchanged three times. The indocyanine green clearance was 0.113. The estimated volume of the future liver remnant was 507 mL and 31%. Percutaneous transhepatic portal embolization of the right portal vein was performed. Two months later, right hepatectomy, extrahepatic duct resection, and portal vein resection were performed (Fig. 1). The left hepatic artery (LHA) was carefully exfoliated to the threshold of the hepatic parenchyma, but the bifurcation of the LHA to the segment 2 artery (A2) plus the segment 3 artery (A3) and segment 4 artery (A4) was not dissected; the tissue surrounding the LHA was difficult to dissect due to the inflammation wrought by preoperative cholangitis. Histological examination of the tumor showed moderately differentiated adenocarcinoma (pathological T4bN1M0, stage IV according to the Union for International Cancer Control classification of malignant tumors, 7th edition [3]).

**Fig. 2:**
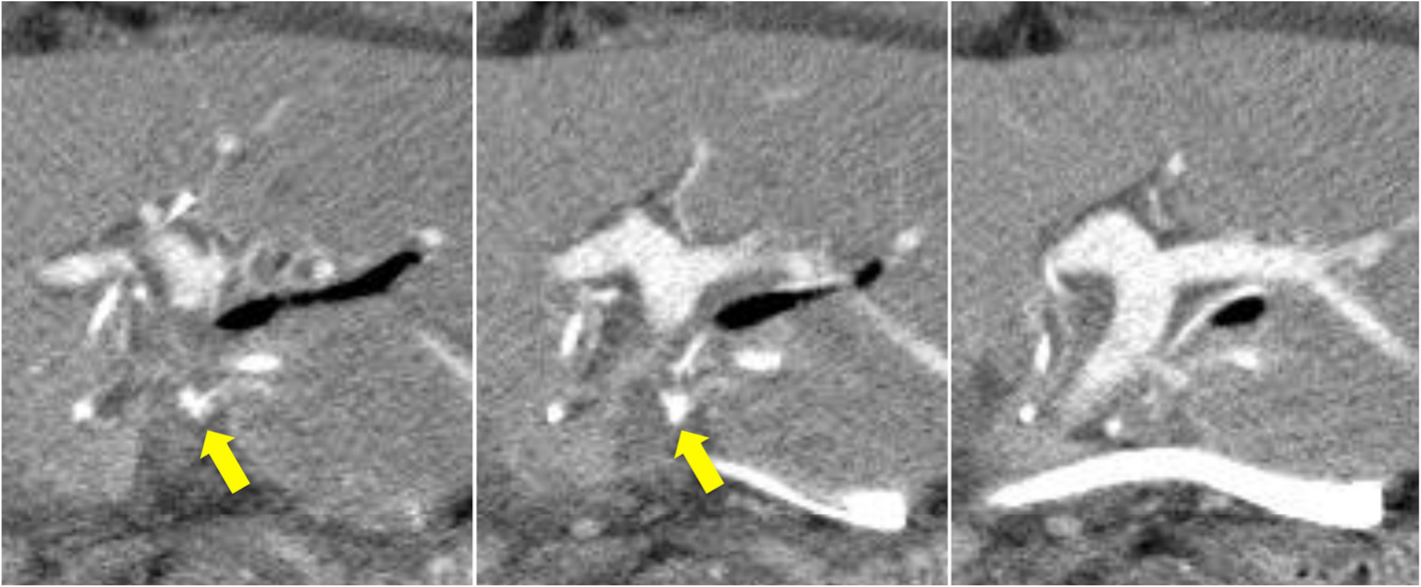
Computed tomography revealing a 6-mm aneurysm of the left hepatic artery on postoperative day 6

**Fig. 3:**
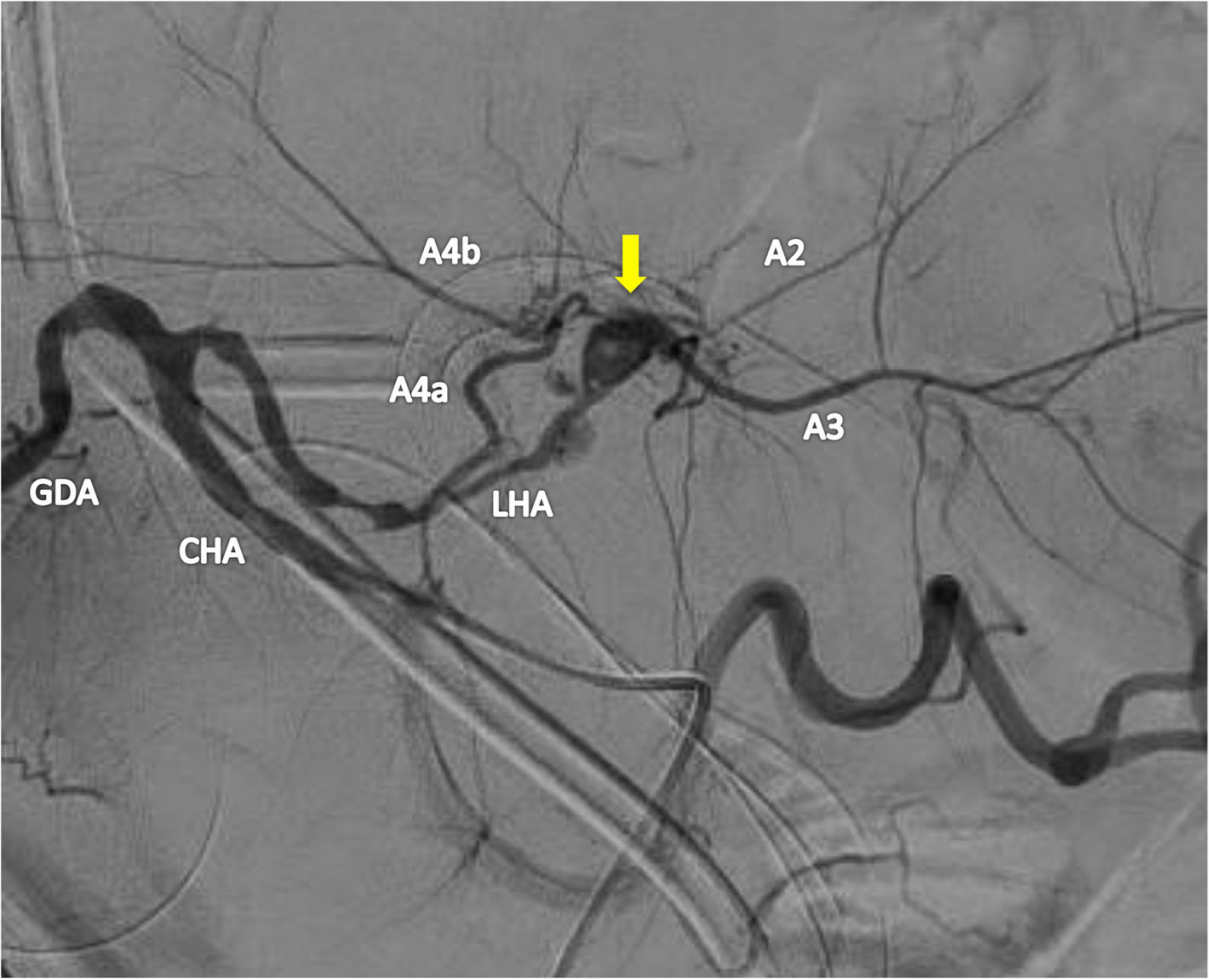
Angiography showing the hepatic artery pseudoaneurysm (arrow) on the bifurcation of the LHA to A2 plus A3 and A4. CHA, common hepatic artery; LHA, left hepatic artery; GDA, gastroduodenal artery; A2, segment 2 artery; A3, segment 3 artery; A4, segment 4 artery

**Fig. 4:**
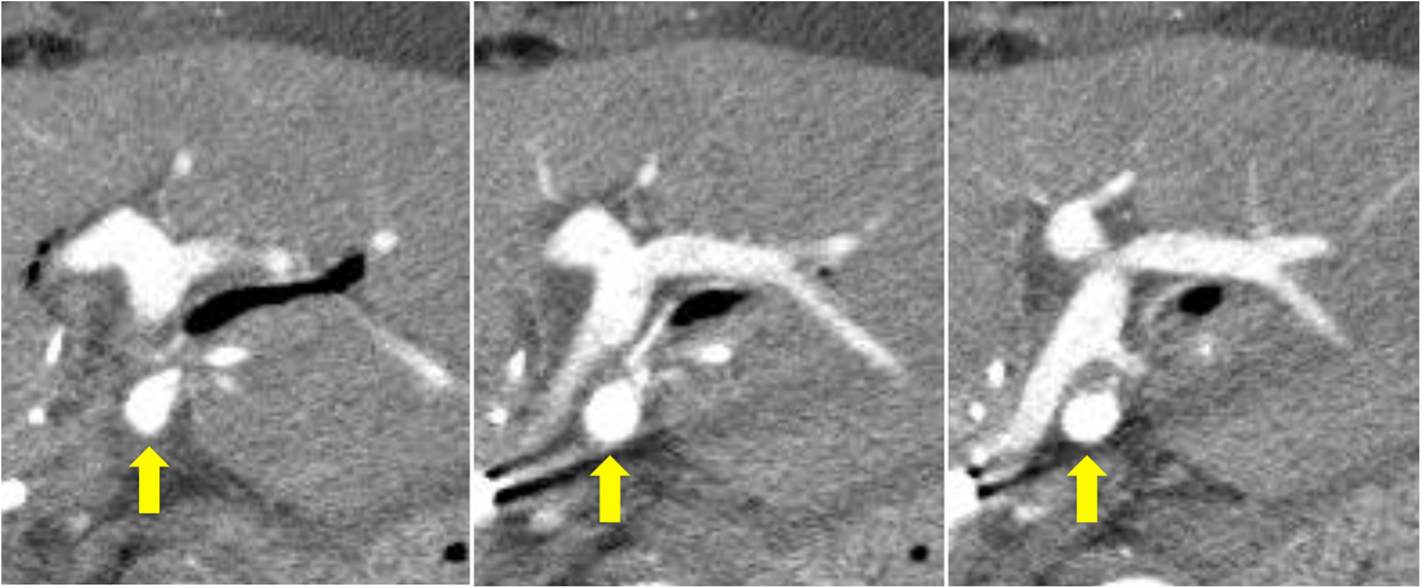
Computed tomography showing enlargement of the pseudoaneurysm (arrow) to a diameter of 10 mm on postoperative day 15

**Fig. 5:**
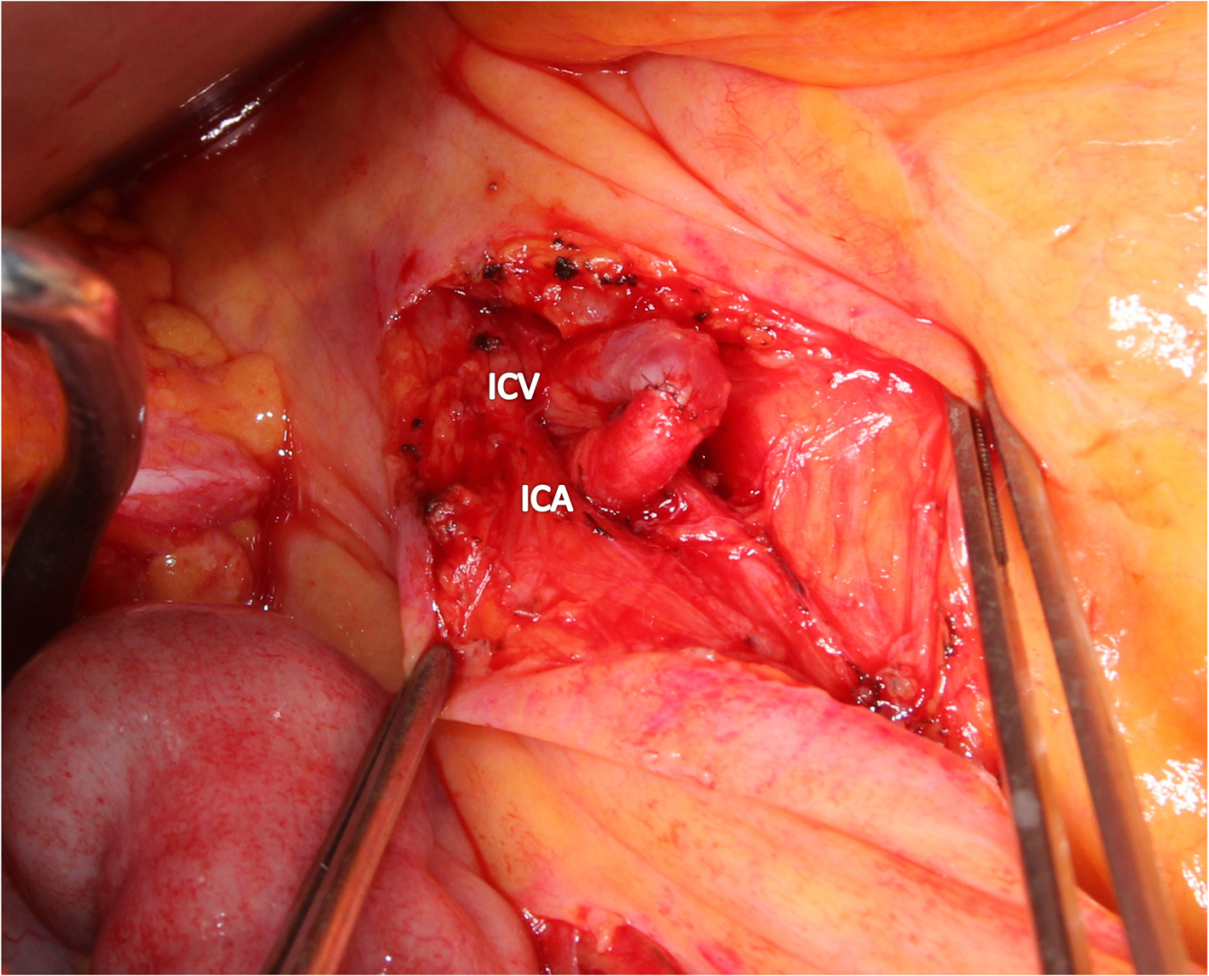
Portal vein arterialization using the ICA and ICV. ICA, ileocolic artery; ICV, ileocolic vein

**Fig. 6:**
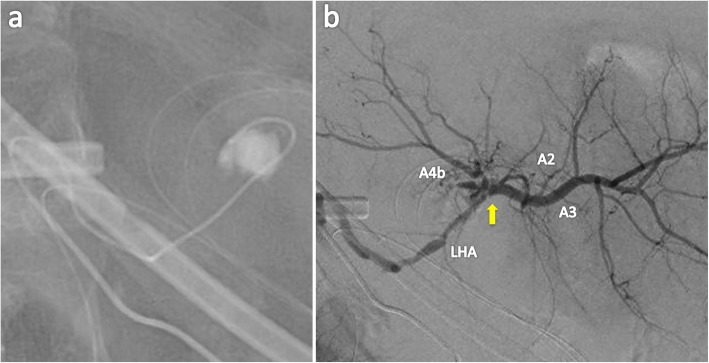
**a** Selective embolization of the pseudoaneurysm with liquid thrombin. **b** Angiography 21 days after embolization of the aneurysm showed patency of the LHA. LHA, left hepatic artery; A2, segment 2 artery; A3, segment 3 artery; A4, segment 4 artery

Postoperative blood examination showed slight elevation of liver enzymes and total bilirubin, and the patient had an uneventful postoperative course without liver failure or bile leakage. Screening CT on postoperative day (POD) 6 revealed a pseudoaneurysm of the LHA with a diameter of 6 mm (Fig. 2). Angiography showed that the sac-like pseudoaneurysm was located on the bifurcation of the LHA to A2 plus A3 and A4 (Fig. 3). Stent placement in the LHA or selective embolization of the pseudoaneurysm was considered for treatment. However, it seemed difficult to place the arterial stent because the LHA was too narrow and the pseudoaneurysm was located very close to the arterial bifurcation. We also hesitated to perform selective embolization because of the higher risk of migration of embolus material to the LHA. Therefore, we carefully followed up the pseudoaneurysm. However, CT on POD 15 showed enlargement of the pseudoaneurysm to a diameter of 10 mm; therefore, we decided to embolize the pseudoaneurysm to prevent rupture (Fig. 4). As a cautionary measure, portal vein arterialization (arterioportal shunting) was planned to maintain the oxygen level of the remnant liver, even if the LHA was occluded by migration of the embolus material. The arterioportal shunt was constructed by anastomosing the ileocecal artery and vein under general anesthesia on POD 15 (Fig. 5). Next day (POD 16), embolization of the pseudoaneurysm was successfully performed by selective injection of liquid thrombin without occlusion of the LHA (Fig. 6a). The postoperative blood data were within normal limits. Refractory ascites (3 L/day) developed thereafter, and portal hypertension was suspected as the major cause of the uncontrollable ascites. Twenty-one days later (POD 37), re-angiography confirmed complete embolization of the pseudoaneurysm and the patency of the LHA (Fig. 6b). Coil embolization of the arterioportal shunt was then successfully performed. The ascites was rapidly resolved and the patient was discharged on POD 45.

## Discussion

This case report highlights two important clinical issues: portal vein arterialization prior to embolization of the aneurysm may be a feasible therapeutic strategy for a pseudoaneurysm that develops after hepatectomy for hepatobiliary malignancy to guarantee arterial inflow to the remnant liver, and early embolization of arterioportal shunting after confirmation of arterial inflow to the liver should be performed to prevent morbidity induced by portal hypertension.

The remnant liver oxygenation must be cautiously considered when an intervention is performed for a pseudoaneurysm after hepatobiliary resection. Previous reports have shown that ligation of the hepatic artery to treat a pseudoaneurysm after hepatectomy results in fatal liver failure [2, 4]. Selective embolization of an aneurysm is an established procedure, but there is a risk of migration of the embolic agent to the hepatic artery, potentially resulting in hepatic parenchyma infarction [5–7]. Although the use of a stent graft for a hepatic artery aneurysm allows maintenance of the hepatic arterial flow, it cannot be attempted for a narrow artery or at a bifurcation of vessels [5, 8–10]. In the present case, the pseudoaneurysm was located close to the bifurcation of the LHA to A2 plus A3 and A4, and the LHA was extremely narrow; therefore, stent graft placement could not be attempted. Embolization of the pseudoaneurysm was associated with a risk of occluding the LHA by migration of the embolus material. Therefore, we performed arterioportal shunting before embolization of the pseudoaneurysm to maintain the oxygen level of the remnant liver, even if the LHA was accidentally occluded. Fortunately, we were able to selectively embolize the aneurysm. We selected liquid thrombin as the embolization material because coil embolization developed halation of CT that made follow-up of the pseudoaneurysm difficult, and n-butyl cyanoacrylate with lipiodol embolization had a possibility to become indistinct of size of the pseudoaneurysm.

The arteriovenous shunt uses the mesenteric vessels and resolves the liver ischemia caused by thrombosis or ligation of the hepatic artery after hepato-biliary-pancreatic surgery or liver transplantation [2, 11–13]. Although previous reports have shown that arterioportal shunting can salvage the totally dearterialized liver and prevent fatal liver failure, Iseki et al. [2] described the risk of reperfusion liver injury caused by arterioportal shunting after ligation of the hepatic artery [13]. Therefore, we performed arterioportal shunting between the ileocolic artery and vein before the interventional radiology. This is the first report of performing arterioportal shunting prior to embolization of a pseudoaneurysm. This procedure would be a feasible therapeutic strategy for a pseudoaneurysm after massive hepatectomy.

Arterioportal shunting is associated with postoperative complications of shunt occlusion and portal hypertension with significant ascites, hyperbilirubinemia, and gastrointestinal bleeding [2, 13]. Bhangui et al. [13] reported that the mortality after arterioportal shunting for the totally dearterialized liver was high, but most causes of death were not related directly to arterioportal shunting because patients were already very severely ill before shunting. They reported that only 1 of 52 patients died from a complication after arterioportal shunting (shunt occlusion and reperfusion injury). Significant ascites, variceal bleeding, hyperbilirubinemia, and liver fibrosis can occur after arterioportal shunting, but the long-term outcomes of arterioportal shunting have been reported to be acceptable when post-embolization management was adequate [2, 13–15]. Our patient also developed refractory ascites associated with portal hypertension, and embolization of the shunt rapidly resolved it without any problem. Thus, early embolization of arterioportal shunting after confirmation of arterial inflow to the liver should be performed to prevent morbidity induced by portal hypertension, and arterioportal shunting should be a temporary and the final option in an emergency situation.

In our patient, the cause of the pseudoaneurysm was not known. Notably, our patient did not develop bile leakage, a pancreatic fistula, or an intra-abdominal abscess; thus, development of the pseudoaneurysm was not influenced by pancreatic juice, bile, or infection. The inflammation around the LHA was severe due to preoperative cholangitis, and dissection of the LHA was difficult. Therefore, we postulate that a retraction injury or thermal injury by the electrocautery device intraoperatively may have occurred.

The patient in our case was asymptomatic, and dynamic CT revealed the pseudoaneurysm. It is vitally important to discover an asymptomatic aneurysm and perform a swift intervention to prevent mortality. Therefore, screening CT or ultrasonography is recommended after major hepatectomy with extrahepatic duct resection to check for the presence of an aneurysm.

## Conclusions

Portal vein arterialization prior to embolization of the aneurysm may be a feasible therapeutic strategy for a pseudoaneurysm that develops after hepatectomy for hepatobiliary malignancy to guarantee arterial inflow to the remnant liver. Early embolization of arterioportal shunting after confirmation of arterial inflow to the liver should be performed to prevent morbidity induced by portal hypertension.

## Data Availability

The datasets used during the current study are available from the corresponding author on reasonable request.

## Abbreviations

A2: Segment 2 artery
A3: Segment 3 artery
A4: Segment 4 artery
CT: Computed tomography
LHA: Left hepatic artery
POD: Postoperative day
